# Stakeholder communication and client understanding of viral load suppression in Blantyre, Malawi—What role did U = U play before the flip the script campaign?

**DOI:** 10.1371/journal.pone.0334337

**Published:** 2025-12-08

**Authors:** Astrid Berner-Rodoreda, Boniface Chione, Esther Ngwira, Rosalia Dambe, Sam Phiri, Till Bärnighausen, Yussif Alhassan, Miriam Taegtmeyer, Petros Tembo, Caryl Feldacker, Christine Kiruthu-Kamamia, Florian Neuhann

**Affiliations:** 1 Heidelberg Institute of Global Health, University of Heidelberg, Heidelberg, Germany; 2 The Lighthouse Trust, Blantyre and Lilongwe, Malawi; 3 Lilongwe University of Agriculture and Natural Resources, Lilongwe, Malawi; 4 Meharry Medical College Global Health and HIV Clinical Services, Blantyre, Malawi; 5 Partners in Hope, Lilongwe, Malawi; 6 Liverpool School of Tropical Medicine, Liverpool, United Kingdom; 7 Department of Global Health, University of Washington, Seattle, United States of America; 8 The United Nations University, Maastricht Economic and Social Research Institute on Innovation and Technology, Maastricht, The Netherlands; SKYDA Health Nigeria, NIGERIA

## Abstract

**Background:**

Various studies have demonstrated the effectiveness of viral load suppression (VLS) in preventing sexual transmission of the human immunodeficiency virus (HIV), leading to the slogan **“U**ndetectable **= U**ntransmittable” or “U = U”. As few studies have examined health stakeholders’ understandings of U = U, the communication of a suppressed viral load (VL) to male clients, and male clients’ understandings of the benefits of a suppressed VL in Sub-Saharan Africa, we explore these in this study. In the field of HIV, it is only in recent years that men who have sex with women (MSW) have been given some attention. The treatment cascade shows that MSW lag behind women in knowing their HIV-status, accessing treatment and suppressing their VL.

**Methods and findings:**

Our findings are based on in-depth qualitative semi-structured in-person interviews with 16 local stakeholders (health facility personnel, NGO and church-based HIV programme implementers and applied researchers) and 39 men on antiretroviral treatment (ART) in Blantyre, Malawi: Twenty-three men with a detectable VL and 16 with a suppressed VL were included in the study. Thematic analysis of the audio-recorded and transcribed interviews show that stakeholders were mostly unfamiliar with the slogan U = U. Despite realizing the advantages for clients and their partners, stakeholders were cautious about how they conveyed information about an undetectable VL: they were worried about clients either misinterpreting an undetectable VL as being cured of HIV or engaging in risky sexual behaviour. This attitude prevailed irrespective of the degree of stakeholders’ endorsement of the evidence of U = U. Male clients were aware of personal health benefits of a suppressed VL, yet unaware of their non-infectiousness. In order not to infect others, some men ceased sexual relations or forwent procreation.

**Conclusion:**

For U = U to become part of a client’s actionable knowledge with regard to treatment benefits, U = U needs to be communicated comprehensively. To what extent recent policy changes, the Flip the Script campaign to make U = U known and staff training programs have led to a different practice will need to be further researched, using our findings as a reference point. Integrated health services are already in place; however, VL monitoring, and testing and treatment for sexually transmitted infections (STIs) should ideally be conducted more frequently than annually, so that Malawian clients can be assured of their VL status and of not passing on the virus while also being able to get any STIs treated.

## Introduction

Informing people living with HIV (PLHIV) that they are non-infectious when their VL is undetectable has improved the health of PLHIV mentally, sexually and through greater adherence and viral control [1]. The message has also underscored the public health benefits of treatment as prevention [2]; in some countries, it led to decriminalizing PLHIV on ART who had not disclosed their status or who had been engaged in unprotected sex [3]. In 2016, the Prevention Access Campaign made the message U = U widely known. The slogan was based on evidence from three large randomized controlled trials. These were: a) HPTN 052 with 1,763 primarily heterosexual discordant couples in Africa, the Americas and Asia [4,5], b) Opposites Attract with 358 discordant men who have sex with men (MSM) couples from Australia, Thailand and Brazil [6], and c) the European PARTNER studies with 548 heterosexual and 380 MSM discordant couples [7,8]. These studies demonstrated no transmission from a virally suppressed PLHIV to their partner [9]. The wording has over the years changed from “a negligible risk” in Switzerland’s early public health statement [2] to “zero risk” of transmission [10]. The World Health Organisation defines a suppressed VL as showing less than 1000 viral copies/ml blood [11], a definition we used for our study. Many countries including Malawi now have VL machines with thresholds of detection as low as 30 and 40 viral copies/ml blood.

The extent to which the scientific evidence expressed in the slogan U = U is known, believed and informs behaviour has been examined in studies among MSM [12–15] and ‘sexual minority men’ [16,17]. Rendina & Parsons noted scepticism among 1/3 of HIV-positive and the majority of HIV-negative participants regarding U = U in a US survey [17]. A few years later, a bigger US survey revealed that the majority of participants felt that U = U exercised a positive effect on their self-perception as HIV-positive sexual minority men; it had also contributed to a reduction of societal stigma [16]. This echoed findings from a study covering 25 countries, where the authors noted better adherence to ART, VLS, and willingness to disclose their HIV status in PLHIV who were informed about U = U by their health care providers [1]. The study thus underscored individual health benefits as well as a decrease in HIV-related stigma. A more recent US study highlighted that 96% of mostly white gay men were aware of U = U but only 41% believed the message; among HIV-negative men, the percentage of those disbelieving the veracity of U = U was even higher [18]. Studies thus indicate that over time, familiarity with the U = U message has increased and seems to have a positive effect on PLHIV, yet many PLHIV and an even greater number of HIV-negative participants seem to remain sceptical of its truth content.

Behaviour based on an understanding of U = U has varied widely: an Australian study found a reduction in sexual activities by PLHIV with undetectable VLs [12]; by contrast, other MSM studies have reported greater risk-taking as a result of a known or perceived suppressed VL [15,19], and men with detectable VLs engaging in unprotected sex [13] or misreporting their VL as suppressed [14].

Relatively few studies on U = U have been conducted in Africa so far [20] despite the fact that the continent has the largest number of PLHIV [21]. Given that U = U is based on the concept of treatment as prevention (TasP) [22], community reactions to TasP can be seen as an indication for the reception of U = U. A study in two Southern African countries revealed that people were somewhat bemused about using antiretroviral drugs (ARVs) to prevent HIV [23]. A South African study showed that awareness of TasP could motivate men to start ART [24], and in Malawi, villagers receiving information on TasP tested for HIV in higher numbers and showed more acceptance toward partners on ART [25].

Globally, fewer than 20% of studies included a provider perspective on U = U [20]. A survey of general practitioners in Australia showed that while almost three-quarters of them accepted the veracity of U = U, only one third discussed U = U with their clients [26]. A qualitative study in Canada, the first country to endorse U = U [27], found that among sexual health care providers working mainly with gay, bisexual and MSM clients, there was little scepticism about the message, yet the extent to which this was communicated to the client was based on the specific provider-client encounter [28]. Conversely, in Kenya, a qualitative study involving health care workers and HIV-negative partners in sero-discordant relationships showed that neither group had much faith in U = U and preferred to promote or maintain other safety measures [29]. A questionnaire-based study with PLHIV in 25 countries, of which South Africa was the only African country, found fewer conversations about U = U between health care workers and MSW (57.6%) than with MSM (70.5%) [1].

MSW are a hitherto neglected group in HIV-related research [30] and in the global and national responses to HIV prevention and treatment [31–33]. MSW have been shown to underutilize health services and to consistently lag behind women in health outcomes in many countries [34–37]. This is also particularly borne out with regard to the HIV treatment cascade of testing, accessing ART and suppressing one’s VL, where men lag behind women by 8–11% [21]. This also held true for Malawi at the time of research [38].

Based on countries’ resources, there is variation in the frequency of VL measurements globally. While countries in the Global North such as the US, Germany and Austria recommend VL monitoring every 3–6 months once viral suppression is sustained [39,40], countries in Sub-Saharan Africa like Malawi, Kenya and South Africa offer VL monitoring on an annual basis once PLHIV suppress their VL [41–43]. An unresolved issue of U = U is how to ensure that there will be no rise in other sexually transmitted infections (STIs) when people know they cannot pass on HIV and do not use condoms for their sexual relationships [44]. The New York guidelines on U = U recommend three monthly screenings for STI for a person on ART [45] – a frequency that may not yet be possible to introduce in resource-poor settings.

Our study with stakeholders (health facility personnel, NGO and church-based HIV programme implementers and applied academics) and men on ART in Malawi thus contributes to the dearth of literature on U = U in Africa. It set out to explore the perceptions of U = U among key HIV stakeholders in Blantyre, Malawi and their communication of a suppressed VL to (male) clients and to examine male clients’ understanding of VL and VLS. We used cognitive dissonance theory for analysing the findings [46].

## Materials and methods

### Study setting and national guidelines

Malawi has a national HIV prevalence of 8.9%, with the South-West and Blantyre City recording the highest level in the country (14.2%) [47]. In the first Malawi Population-Based impact assessment (MPHIA), Blantyre City registered the lowest national VLS at 59.5% [48]; this increased to 81% in the second MPHIA [47]. Malawi is now one of seven countries that has already reached the UNAIDS treatment targets for 2025 [21].

According to Malawi’s national HIV treatment guidelines, PLHIV should start on ART as soon as they have tested positive and receive a VL test after six months on ART and then in two-year intervals [49]. From 2019, annual VL tests and six-months’ supplies of ARVs to clients with a suppressed VL were introduced (<40 copies/ml or low detectable levels); this was for all clients who had been on ART for at least six months and had experienced no side-effects or opportunistic infections [41,50]. Upon ART initiation, PLHIV receive group and individual adherence counselling [49]. If their VL remains detectable after six months on ART, they undergo additional intensive adherence counselling and are given either monthly or a three-months’ supply of ARVs. After three months, another VL test is taken [50]. Recently, Malawi developed its own vernacular version of U = U. The Chichewa version “T = T” (Tizirombo Tochepa = Thanzi) [51] literally means “few viruses/bugs equals health”. The Malawian rendering thus focuses on health rather than on non-infectiousness. The private-public-US-funded Flip the Script campaign developed tools for communicating U = U together with health care providers and ART champions in Malawi and Zimbabwe in collaboration with the respective governments [52]. These developments and trainings of some health care providers occurred after our data had been collected.

In late 2019, Heidelberg University and the Lighthouse Trust conducted a mixed methods study on men and ART to a) examine factors related to VLS at the Umodzi Family Centre (UFC), a Lighthouse Trust operated ART facility in Blantyre and b) to better understand men’s experience and issues with ART, ART service delivery and VLS from various perspectives. We carried out in-depth interviews with men on ART, stakeholders and men in the community, and we conducted participant observation in the Blantyre District. The overall study was primarily carried out at UFC, a facility that integrates HIV services with TB, NCD, STI and family planning. It is affiliated to Queen Elizabeth Central Hospital, which is a tertiary referral and teaching hospital in the southern region of Malawi. In 2022 (after a COVID-19 study interruption) more observations were made at the clinic and design-thinking workshops were conducted with men to develop prototypes of tailored ART service provision.

### Study design and sampling

This qualitative study on U = U, VL communication and understanding is nested within the larger mixed methods study on men and ART in Blantyre district as explained above. The study presented here is based on in-depth interviews with key HIV stakeholders and men on ART at UFC.

We purposively selected local stakeholders with direct experience of men utilizing HIV/ART services by drawing up a list of 20 potential stakeholders based on the recommendation of the local partner organisation, local and international NGOs and our own research on key local players. All potential stakeholders were contacted by phone or visited prior to the interview to ensure that they had experience with male clients and were interested in participating in the study. Stakeholders included health care providers (clinicians, clinical officers, nurses, counsellors) of the partner organisation and other health providers in the Blantyre district, local HIV researchers involved in HIV programme and non-state church-based and secular implementers.

Men on ART at UFC with an age range of 18–50 + years and at least one VL test result within the last 3–4 months were identified as potential study participants. UFC staff in collaboration with local research assistants contacted and scheduled interviews with potential male participants (with undetectable as well as detectable VLs). We followed the qualitative research guidance to stop interviewing when saturation has been reached [53,54].

### Data collection

Recruitment for the interviews started on August 19^th^, 2019 and was completed by December 6^th^, 2019. Between November and December 2019, we conducted in-depth semi-structured interviews with study participants. Participant observation, practiced in both the waiting area at UFC in late 2019 and in consultation rooms in 2022, provided further information on interactions between health personnel and clients.

Each interview lasted approximately 30–60 minutes. The first author, an experienced qualitative researcher trained local research assistants on the study protocol, ethical requirements and qualitative research methods prior to data collection. Interviews were held in either English or Chichewa according to the preference of the interviewee.

Before data collection commenced, the research team presented the study to health care providers at UFC and discussed whether their clients could be asked about U = U. Health personnel were wary of approaching men on this issue, as they feared that men might misunderstand the message. Our research team therefore explored the slogan with stakeholders only, but explored men’s understanding of VL and VLS as this would tell us what benefits they saw in a suppressed VL.

Semi-structured interview guides were piloted in late August 2019 and adapted as the interviews evolved. The interview guides for stakeholders and men on ART explored the following key domains: Both stakeholders and men on ART were asked about the perception of HIV and ART in communities, men’s enablers and challenges of taking ART, and their vision of male-friendly ART service delivery. Men on ART were additionally asked how they found out about their HIV status, how they were initiated into ART and experienced ART services, while stakeholders were additionally asked about testing approaches for men and their understanding of U = U in relation to male ART clients. The research team held daily debrief sessions during data collection to discuss and reflect on the data, the interview experience and to identify data gaps [55]. This led to more probing on pertinent issues such as men’s understanding of VLS. In this article we focus on U = U and the understanding and communication of VLS.

### Data analysis

All interviews were, with the permission of interviewees, audio-recorded and transcribed manually and verbatim; interviews conducted in Chichewa were translated and transcribed simultaneously. Four transcribers simultaneously translated and transcribed primarily interviews conducted in Chichewa and one transcribed interviews conducted in English. All transcripts were reviewed for completeness and accuracy by the research team. Transcripts were checked against the audio recording by the first author; transcripts translated from Chichewa were additionally checked by the third author. In cases of divergence to the recording, transcripts were corrected to capture verbatim what the interviewee had said.

The first author coded the interviews in NVivo Pro 12 based partly on topics of the interview guides. These included codes, such as “unfamiliar with U = U” as well as issues identified in the interview data [54], such as “refraining from sex”, thus combining deductive and inductive approaches, as is common in qualitative interview research in Global Health. Transcripts were re-coded as more codes were added. Co-author YA coded 10% of the interviews to enrich the understanding of the transcripts. In line with Braun and Clarke [56] and Smith and McGannon [57], we view the main benefit of co-coding and jointly interpreting qualitative data in an enhanced understanding of the data that will increase depth, rather than in establishing intercoder-reliability. Our thematic analysis [58,59] was based on familiarization with the material through transcribing, reading and re-reading the transcripts, coding and discussing and determining themes. We used triangulation in comparing and contrasting responses within each respondent group, across respondent groups and between interviews and observations. We discussed interview contents in the team to identify the following key themes and their interconnectedness within and across interviews [58,59]: a) the stakeholders’ beliefs and understanding of U = U; b) the stakeholders’ uncertainty about how to communicate both U = U and a suppressed VL without this leading to misunderstandings among clients; and c) male clients’ understanding of VL and VLS.

### Ethical considerations

Ethical approval for the study was obtained from the National Health Sciences Research Committee (NHSRC) in Malawi (#19/05/2336) and the Heidelberg Ethics Commission in Germany (S-219/2019). All researchers successfully completed a FHI 360 online course on research ethics prior to data collection to ensure that the study was conducted to the highest ethical standards.

All interview participants were above 18 years of age and provided voluntary written informed consent prior to the interview after having been informed (orally and in writing) of the purpose of the study. The interviewer witnessed the written informed consent. All interview participants received compensation for their time and travel expenses, equivalent to US$ 10, as required by NHSRC.

To protect the interviewee’s identity, all interviewees were given pseudonyms (codes) consisting of IDI for in-depth interview, a letter from the start of the alphabet indicating the respondent group, the interviewer’s initials, the number of interviews conducted on that day by the same interviewer, and the date. All data was uploaded and stored in a password protected and safe cloud through the research institute to which only the research team could gain access. Audio-recordings were deleted after a quality-check of the transcript had been made.

## Results

### Participant characteristics

Our results are based on 16 interviews with stakeholders and 39 interviews with men on ART (23 with unsuppressed, 16 with suppressed VLs), see Table 1.

**Table 1:** Respondent Table.

Respondent Groups	Male (n = 68)	Female (n = 4)	Median Age [age range]	Highest Educational Level Primary Secondary Tertiary		
**Men on ART with a suppressed VL**	16		35 [18-54]	4	10	2
**Men on ART with a detectable VL**	23		34 [18-53]	5	13	5
**Stakeholders**	12	4	44 [26-61]	0	1	15

Stakeholders consisted of applied researchers, implementers (NGO and church-based), and employees of health facilities in the Blantyre District. They had a median age of 44 years and were predominantly educated at tertiary level (n = 15). Four stakeholders were female. The median age of men on ART with VLS was 35 years, men with detectable VLs had a median age of 34 years. Men on ART in our sample were mostly educated at secondary level (n = 23).

**Fig 1:**
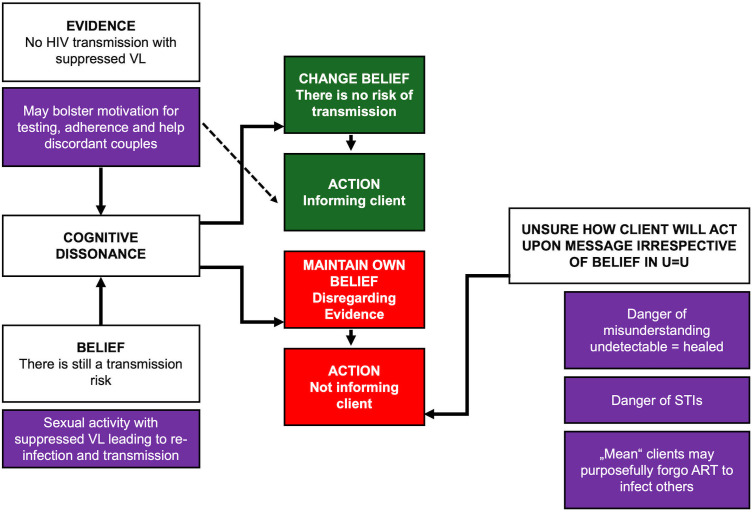
Stakeholders’ U = U considerations based on an amended cognitive dissonance model.

We present stakeholders’ U = U considerations based on an expanded model of cognitive dissonance [46], see Fig 1. We start with stakeholders’ own beliefs and understanding of U = U. The dotted arrowed line indicates that even when stakeholders endorsed the evidence of U = U, there was no automatism in talking about non-infectiousness to clients. We extended the model to include stakeholder perceptions of likely client responses. The envisaged client responses and actions informed stakeholders’ willingness to tell clients about U = U, irrespective of whether stakeholders endorsed the slogan. This is captured in the section “Uncertainty how to communicate the message without causing harm”. In a third section, we present the way men on ART received and understood information about a suppressed VL.

### Stakeholders’ beliefs and understanding of U = U

Few stakeholders had heard of the slogan U = U. No stakeholder working in a health facility in our sample was initially familiar with the slogan. When it was explained, the reaction by many stakeholders across respondent sub-groups was ambiguous: they saw positive aspects but also expressed reservations. Among the few who were familiar with U = U and promoted it privately or through their work, one mentioned a church meeting: “I was talking to them on this issue of viral load that if your viral load is undetectable, you cannot transmit the virus. Everybody goes: ‘What??’” (stakeholder 1, research). The fact that scientific evidence changed over time was seen as contributing to the confusion.

I think the messaging that has been there before to say, if you are HIV-positive, you don’t use a condom, chances are, you would transmit the infection regardless of the fact that you are on ART... So, now that we have got like more of the evidence coming, yeah like u = u, yeah, I know, the community members that I have met so far in those meetings when you introduce this topic, most of them would not believe it…. But the message we are telling them is to say: No, I think this is the evidence because it’s not based on one study, there are actually several studies that have been conducted and the results have actually shown that when you are undetectable, your viral load is undetectable, there are actually no chances that you can transmit the infection, yeah? (stakeholder 3, research)

For stakeholder 3, the fact that several studies confirmed the evidence was important in popularizing the message. Stakeholders perceived U = U as liberating for men in forgoing condom use while still “prevent(ing) transmission to their loved ones, to their wives and others” (stakeholder 12, health facility). The message was seen as “low hanging fruits” for serodiscordant couples (stakeholder 13, NGO-based HIV work).

you can’t tell them to use condoms for life. They don’t. In fact, they don’t. To me, it was like: that’s the better message to tell them. This is a discordant relationship, you need to be on treatment and achieve viral suppression as quickly as possible. You can stay in the relationship without requiring condoms on every day, you will still be discordant and emphasizing the importance of treatment and achieving viral suppression to the infected partner. (stakeholder 13, NGO-based HIV work)

Yet, stakeholders self-critically assessed that these benefits were not widely known because they had not spread the message.

Yeah, that message U = U also needs to be, how should I say, yeah, we need to popularise the message because it’s only us who are within the regime [on ART] to say, if you stay on a medication for a long time, observe your times, observe your other characters that you do, then your viral load is going to be suppressed, then you cannot pass on to your wife or your girlfriend. It’s only those who are within that, though some still don’t know. (stakeholder 4, church-based HIV work)

Benefits were perceived for PLHIV suppressing the VL, their partner(s) and for vertical transmission.

it’s like when you have HIV with low or undetectable viral load, the transmission rate is reduced, so your partner has less chances of getting infection. So, likewise, also if another person has also a low detectable viral load, to you as well the transmission rate risk is low, so we can see that it is of benefit. As well as a mother who has an undetectable viral load, transmission to the baby is also minimized. (stakeholder 7, health facility)

While this stakeholder acknowledged the benefits of U = U, he expressed it in more cautious language (reduced and low risk) rather than endorsing “zero” risk. Additional benefits were seen in motivating couples to test for HIV as this would have advantages for both of them.

Say, for example, you have got a girlfriend, so you would be eager to know their status because you know that if they are HIV-positive, you would encourage them to take treatment because you know that that treatment is also going to help you, because if their viral load is undetectable, then for sure you are protected. (stakeholder 3, research)

Bolstering adherence was viewed by many as another positive side-effect of U = U.

I think this can encourage them to be on track in as far as taking [antiretroviral] drugs is concerned because now, they would know that I will reach a point or a situation where after taking the medication faithfully, I wouldn’t transmit it. (stakeholder 15, church-based HIV work)

The greatest advantages of U = U across the stakeholder group were seen in easing the life of discordant couples and improving health by bolstering adherence: “they are taking their medication correctly, and the outcomes are good” (stakeholder 11, NGO-based HIV work). On a societal level, U = U could even lead to epidemic control. “If you don’t transmit the virus to your partner, it means you are controlling the epidemic. At a certain stage we will have an HIV-free generation because there will not be transmissions” (stakeholder 5, health facility). Almost all stakeholders saw the appeal of the slogan U = U for all sexually active men and in particular for men spending extended periods away from their families (soldiers, fishermen, truck-drivers).

Despite these perceived advantages, many stakeholders also voiced reservations or disbelief combined with fears of men increasing their sexual activities. “Because they may think that just because I have an undetectable viral load, then I can get engaged in any promiscuous behaviours and then, there might be some chances to transmit the infection to others” (stakeholder 11, NGO-based HIV work). A number of stakeholders, among them health personnel, upheld condom usage: “they [male clients] have to continue practicing self-principles like barrier during sexual activities” (stakeholder 16, health facility), and emphasized the need to consult with clients along these lines.

So, they think, ah, … maybe why don’t I have sexual intercourse without using a condom because it’s like, I’m alright, yeah so they would quickly jump into thinking of that. Yeah, so that’s why you still have got to take more time in chatting with them telling them the right information. (stakeholder 8, health facility)

This position was maintained even by those who felt the risk was negligible:

the message we need to be giving to our clients is that they still have a chance of transmitting. Of course once the viral load is low, the chance is very low that they can transmit. But they still need to be using the condoms. (stakeholder 9, health facility)

A stakeholder who had emphasized the need for the message of U = U to be spread in the community expressed caution when it came to child-bearing and referred the question to experts:

most of the times the question that comes, can I be able now to have a child? So that aspect, mostly we should leave it with the medical personnel rather than us as HIV activists trying to explain the benefits of being suppressed with a low viral load or even to say now you can have sex without a condom, you cannot transmit HIV. To me, I feel, it can be really confusing… (stakeholder 4, church-based HIV work)

Some also highlighted the risk of becoming re-infected with other viral strains, thus doubting not so much the non-infectiousness but the pre-exposure prophylaxis function of ART. One stakeholder in particular was not in favour of making the U = U message known to men:

I think, it can bring negative consequences for the men. They can end up re-infecting themselves with new viruses and now there will be cross-pollination of viruses, and then there will be re-multiplication of viruses, and even more catastrophic consequences. (stakeholder 2, NGO-based HIV work)

Individual beliefs, perhaps mixed with one’s own moral understanding and internalized previous HIV and ART messages, thus counteracted the message of U = U. One stakeholder resolved this tension by leaving the decision to the client, even though the message was not directly shared with the client.

Uhm, there might be some misconceptions, like to say, once you are undetectable then you can’t transmit. Of course, through some abstract that people have written, it has indicated that it’s really true and, of course, I have chatted with some few men concerning them being LDL [low detectable level]. I was asking them like, “if your viral load is undetectable, would you choose to have a baby?” So, one would say: “Ah, no, I don’t see a reason, because I don’t know, maybe the virus is still in, so I might transmit to my partner”, something like that. Yeah, but it’s just about the personal perception on how you see it. So that’s the way I figure it out to say it is based on personal perception. (stakeholder 10, health facility)

While the slogan was seen as a potentially encouraging message for men in terms of boosting adherence and preventing onward transmission, many stakeholders seemed to only partially endorse the message.

### Uncertainty of how to communicate the message without causing harm

Across the board and irrespective of whether U = U was fully or only partially endorsed by stake-holders, they expressed the fear of the message being misunderstood by the client. One consideration was that a one-off VLS might be misinterpreted as being suppressed for life or even as being healed.

Yah, because others would think once the viral load, it means that I can’t transmit the virus anymore, so we need to make sure that we are explaining that. What we are talking about is undetectable viral load because you can remain undetectable to a certain extent. Because if you stop taking ARVs, your viral load can start to go up. So that’s what I am trying to say that we need to make sure that the information gets out properly that it’s as long as your viral load is undetectable not like just once when I went for a viral load test it was undetectable, then you stop even taking ARVs and you think that you remain somebody who can’t transmit the virus. So we need to make sure that information is given properly. (stakeholder 1, research)

A clinical officer talked about the difficulty of translating ‘suppressed’ or ‘undetectable’ into Chichewa where the connotation might be different: “in vernacular, it can mean like ‘cure’”. (stakeholder 7, health facility). Stakeholders worried that men on ART might discontinue treatment if they learnt about U = U. “So they should not relax to say: ‘Ah, it’s undetectable, meaning I am okay’ and then they start skipping their medication and what” (stakeholder 3, research). The fear of clients defaulting led to hesitancy among stakeholders. “Since we have lots of people that are eager to stop taking ART, I think that slogan… it might be misleading.” (stakeholder 16, health facility).

Understanding the term ‘suppressed’ as ‘healed’ might also bolster a belief in ‘faith healing’ which was already perceived as a problem for adherence to ART as some churches were encouraging their members to throw away their ARVs. U = U might lead to more confusion in this setting:

They [Pastors] say, ‘I have prayed for you, stop taking medication….Then go for a test’. Then one day I go to hospital, even some of the medical people will say: ‘well, we do not see any virus.’ That means I am healed, so it’s a testimony to the pastor to say: ‘stop taking medication’. (stakeholder 4, church-based HIV work)

Stakeholders faced the additional problem of documenting and informing the client of a suppressed VL without suggesting that the client was healed. Health personnel noted that men on ART were likely to draw their own conclusions.

Previously, the viral load, the documentation was sometimes, they would say ‘zero’. And once you document and tell the client it is 0, they were like jumping up to say: ‘The HIV is gone.’ So,… we thought: ‘oh, the documentation shouldn’t be 0.’ (stakeholder 8, health personnel)

Some stakeholders used the metaphor of a soldier fighting the enemy (virus) in communicating a suppressed VL to ensure that clients do not stop ART. Further considerations in communicating U = U related to the risk of contracting sexually transmitted infections (STIs). One of the few stakeholders who spread the U = U message recommended certain modifications:

in U = U, chances also are that because you are on ART and you are adherent, so ART on its own, is also like a prophylaxis like would help you to prevent secondary infection, yeah? But we are emphasising to say for like a sexual partner that is not your regular partner, you would not take chances, yeah? Because you know there are chances that you could get like an STI or what have you (stakeholder 3, research)

Engagement in sexual activities without the use of a condom was something stakeholders felt needed to be guarded against. For many, as we have seen before, this was linked to a fear of transmitting the virus; for others, it was linked to an increase in STIs. Moral considerations may have also played a role in stakeholders views of what to tell the client. Two female stakeholders expressed the concern that some men were ‘ill-minded’ (stakeholder 1, research) or ‘bad-hearted’ (stakeholder 16, health facility) and would want to infect others; they would not remain ART adherent, if they knew that they were non-infectious. In other words, they would make sure to maintain a detectable VL in order to infect the partner or others.

Stakeholders viewed the inconsistent taking of ART, stopping ART, and showing resistance to ART medication as leading to a higher VL. Two stakeholders also mentioned unprotected sexual activities: “it’s about missing doses, unprotected sex – yah, those are the major ones.” (stakeholder 10, health facility). This message was also communicated to clients, as we will see in the next section. One stakeholder felt clients should be informed in stages so that they receive the full picture without misunderstanding the message.

especially for those who are on medication, they really need to know that it’s much better to stay faithful on medication so that your CD4 count remains high and your viral load goes down and then that’s where it should not be in one package. The second package will now mean: what does undetectable mean? Then we start explaining to them that undetectable does not mean healed, it means abcd. So it should be a two package… so that they do not confuse the message. (stakeholder 4, church-based HIV work)

### Men on ART’s understanding of viral load and viral load suppression

All men on ART in this study had at least one VL taken, yet their understanding of VL varied: some were not familiar with the term and declared they were never informed about their VL.

I was told to give my blood so that they can do the viral load test. But no one explained what a viral load count is, what are its benefits and drawbacks such that I really do not understand what its benefits are. (man with suppressed VL, 36 yrs)

Some stakeholders pointed out that men are often reticent to ask for information if they have not understood something. This could be noted in practising participant observation during consultancies at UFC when clients received their medication. Very few men asked questions. In the interviews, many men perceived their VL result as an indicator for their adherence to ART and a motivation to take ARVs more consistently. “It helps me to be taking medication as recommended by the doctors” (man with detectable VL, 50 yrs). Men described their VL in terms of ‘low’/‘okay’ or ‘high’; few men and predominantly those below 35 in our sample cited their exact results: “They had found out that it was 1015” (man with detectable VL, 18 yrs). Men’s responses to having an undetectable VL varied. They ranged from stopping ART altogether, as they felt ‘back to normal’, to continuing with ART to keep suppressing the virus.

I felt like my life is back to the normal base I was before, because I was jogging the way I did before. I got my energy back and I was like, even if I don’t take the medicine today, it’s okay…[chuckling] Even when my wife asks me if I have taken the medicine, I would say “yes”, but in a true sense, I hadn’t. But then the health providers, they knew that this person from when we last gave him his dosage and the dates so far aren’t matching, so they called me, telling me that I am missing the drugs, and I accepted. (man with detectable VL, 39 yrs)

Another client seemed to confuse his undetectable VL with his HIV status.

When they tested me, they told me that I don’t have the virus and then I was thinking how could this be possible to be HIV-negative and when I came back I realized that this happens to a lot of people when they are adhering to the prescriptions. (…) I was very happy because if I was found HIV-negative, my friend would have known that I am HIV-positive but that encouraged me to keep on taking medication seriously so that the viruses should not have an opportunity to rise up. (man with suppressed VL, 18 yrs)

The confusion between having a suppressed VL and a negative HIV result reflects stakeholders’ concerns about misunderstanding the U = U message.

Men on ART were of the view that a high VL resulted from poor adherence to ART. Some also linked it to their general health behaviour or to engagement in unprotected sex: “We were told that if we intensify on having unprotected sex with multiple sex partners, it would increase the number of viruses in our body” (man with suppressed VL, 39 yrs). Another man reasoned that a high VL could be “because I have taken alcohol. I also happen to have sex with other people which might increase my viral load” (man with detectable VL, 31 yrs).

None of the men on ART interviewed at UFC talked about the benefits of not transmitting the virus to others. The knowledge of being able to beget children without worrying about transmitting the virus to the partner or the child when their VL was undetectable did not seem widely known.

R: For me as a person, in the name of God, I have never asked any girl to go somewhere with me. What stops me really is the fact that I fear that if by chance I accept one girl and have sex with her, the baby that will come as our gift will have the same problem like mine. So I fear for this child to go through what I have gone through the whole of his life. Sometimes I keep quiet and think about it. But there will come a time when I will ask the doctor, if it is possible for someone like me with HIV to have sex with a girl and have a baby that has no virus. That’s what dwells in my mind the most. That’s why I don’t bother much about being with girls. I simply just chat with them, and life goes on. It is not that I don’t have feelings for the girls. I have the feelings but for me to say, let’s go and have sex, aaah, I am always afraid. Even in my residential area people question as to why I am not interested with girls.

I: Where does your fear dwell really?

R: On the baby. I fear that if I am unfortunate, that the girl I sleep with gets pregnant, the baby will suffer like me the whole of his life. (man with suppressed VL, 29 yrs)

Unawareness of the benefits of a suppressed VL in terms of non-infectiousness could lead to anxiety in relationships: for this 29-year-old man, the concern about not passing on the virus barred him from engaging in sexual relations. Conversely, another 29-year old man with a detectable VL occasionally skipped ART for better sexual performance as he felt that ART sapped his sexual energy.

Few men on ART mentioned the experience or possibility of having an HIV-negative child. They were also perplexed about serodiscordance. “I was found HIV-positive but she was found negative. And the child I gave birth to, is also HIV-negative… That is God’s will because there is time for everything” (man with detectable VL, 38 yrs). Some married men thought it too risky to engage in childbearing: “In my case, I had advised my wife to stop taking an active role in reproduction, to ensure we remain healthy whilst taking treatment” (man with suppressed VL, 45 yrs). Thus, the benefits of an undetectable VL and U = U for relationships and families (in enabling partners to have unprotected sex and to give birth to HIV-negative children) did not generally seem to be known by men on ART.

## Discussion

Our findings reveal that stakeholders in a high HIV-prevalence setting in Malawi were largely unfamiliar with the slogan U = U. Many stakeholders perceived the U = U message as (potentially) beneficial for men, their partners and society at large. Yet they expressed ambiguity and highlighted concerns regarding clients possibly misunderstanding an undetectable VL as meaning they were ‘healed’ and as giving them cause to cease ART. Stakeholders also feared that men would become ‘promiscuous’, infect others and re-infect themselves with different HIV strains or with STIs. They were therefore cautious about how they communicated a suppressed VL, and mostly decided to leave clients in the dark about the benefit of non-infectiousness.

Our amended cognitive dissonance model presents the dilemmas HIV stakeholders faced at two levels: 1) They experienced conflict between the evidence of U = U (no transmission risk) versus their own beliefs (transmission risk still exists). This dissonance seemed to dissuade stakeholders from communicating that a suppressed VL makes the person non-infectious. 2) All stakeholders seemed to grapple with how best to communicate the message in order to avoid causing harm to clients who might stop ART, believing that they were now cured. The second dilemma seemed independent of any dissonance or concordance of evidence and beliefs.

### Stakeholders’ hesitancy in communicating U = U and implications for action

Our findings show that service providers in a high-prevalence setting had by and large not heard of the slogan U = U. When the slogan and the concept were explained, many expressed reservations about communicating it. This tallies with the findings of a systematic review that also included the Sub-Saharan African region [20]. The caution and ambiguity of Malawian stakeholders in explaining the full benefits of an undetectable VL to clients is consistent with similar observations made among health workers in Kenya [29] and studies covering multiple countries [1,60]. Stakeholders in Malawi tended to cling onto earlier messages that highlighted risks of re-infection and transmission. Some viewed condomless sexual activities as the reason for a high VL. The continued emphasis on safe sex has been echoed in other African U = U studies [29,61]. However, the additional risk mentioned by some stakeholders of possibly increasing STIs in PLHIV is a risk that an undetectable VL cannot eliminate; such a risk is not often mentioned in U = U studies. STIs in the context of U = U should be monitored more closely [62].

An important first step would be to train not only health personnel but also other HIV service providers in order to increase understanding about the veracity of U = U so that the message is spread without reservations. This is in line with recent Round Table recommendations from the 12^th^ International AIDS Society Conference in Brisbane [63]. The Malawian T = T strategy [51], a vernacular adaptation of U = U, foresaw training for health personnel between the years 2022 and 2025. To what extent the recent “Flip the Script” Campaign in Zimbabwe and Malawi [52], aimed at popularizing the message of U = U, was able to change attitudes will have to be examined in further research.

An appropriate way of communicating an undetectable VL was a related major concern expressed by stakeholders. The use of the soldier metaphor was an attempt to explain the concept to clients who had received little education. In our sample, younger men below the age of 35 seemed to have a deeper understanding of VL and VLS. While our study was clearly not a population representative sample, the age, gender and education levels of clients should be explored further in U = U studies. Male clients in our study tended not to seek clarification from health personnel, thereby putting the onus on the stakeholder to volunteer information. Stakeholders’ wishes to tailor VL information to clients is a solution that has also been proposed in an Australian study for “culturally diverse groups and people with low health literacy” [12] and a study in Hongkong where lower education and older age were correlated with lower levels of acquaintance with U = U and higher stigma towards PLHIV [64]. Conveying the benefits of VLS, TasP and U = U to clients in a gender-, age- and education-appropriate manner and incorporating new tools (such as apps with video testimonials of PLHIV with a suppressed VL) and Q&As with health personnel on U = U [65], could be a way to educate both health care providers and the community. This would also help to ensure that a suppressed VL is not misunderstood as meaning one has been healed.

U = U should work everywhere and not just in high-income countries (HICs) with frequent VL testing. Yet, the reality of clients in low- and middle-income countries (LMICs) is different. Malawi increased VL testing from once every two years to once a year [41,50]. The concern of Malawian stakeholders that a test many months ago may not inform clients about their current VL status should be heeded. While there is no evidence of transmission to an HIV-negative partner even for those with low viremia (<600 viral copies per mL blood) [66], clients need to know their VL more frequently than once a year in order to be sure that they suppress their VL and cannot transmit the virus to their partner. Even in HICs such as Canada, unequal access to ART services is seen as a drawback for realizing U = U [28]. Increasing the frequency of VL testing also presupposes diagnostic capacity or rolling out point-of-care VL machines to local health facilities and ensuring a short turn-around time for results [29].

The current Malawian treatment guidelines recommend to “explain the benefits of immediate ART for the patient’s own health, and for prevention of onward transmission to sexual partners and from mother to child” [41], and the national HIV Strategy acknowledges no onward transmission with an undetectable VL [67]. At the time of research, this was not clearly spelt out and no further teaching aids existed for health care workers and implementers. The extent to which stakeholders can now draw on materials for facilitating communication with clients or were/are able to receive trainings on U = U will be explored in a follow-on study.

### Possible consequences of not informing about U = U

The fact that men in the Malawi study were aware of the health benefits of ART but were unaware of being non-infectious with a suppressed VL aligns with studies in South Africa [24,68]. These findings call for more treatment literacy for male clients. Our study revealed that men were not only unaware of U = U, irrespective of their VL status, some also held misconceptions on the danger of transmitting the virus, which led them to restrict their sexual interactions, and in one case to refrain from sexual relations altogether. Similar restrictive sexual behaviours in terms of “sexual inactivity” and “reduced frequency of sex” were reported in connection with a poor understanding of U = U in an Australian study [12]. As the national T = T message puts the focus on the health benefit of an undetectable VL rather than on non-infectiousness, future studies will have to investigate if this has led to changes in how stakeholders communicate the benefits of an undetectable VL.

Studies also highlighted the association of a belief in U = U with less self-stigma of PLHIV in South Africa [69] and fewer discriminating enactments against PLHIV by HIV-negative persons in the US [18]. Village TasP education in Malawi has been shown to lead to a greater understanding of non-infectiousness coupled with stigma reduction and an increase in HIV-testing at a community level [25]. Benefits of U = U in terms of stigma reduction, ease of mind in having unprotected sex with one’s partner, begetting children naturally without the risk of onward transmission, and the motivation to adhere to ART, and reducing HIV incidence have been documented elsewhere [60,70]. Stakeholders in Malawi mentioned many of these benefits; however, stigma reduction was only touched upon indirectly in highlighting that it may help discordant couples. This is despite the fact that community stigma and anticipated stigma by PLHIV was a prevalent issue in the qualitative study [71], yet stigma was not highlighted by stakeholders (ibid). In being less aware of the stigma dimension that PLHIV faced, the benefits of reducing stigma may also not be foremost in stakeholders’ minds. Not communicating the U = U message can thus be seen as a missed opportunity to reduce self-stigma as well as societal stigma towards PLHIV.

## Conclusion

Our study highlights that, irrespective of whether stakeholders in Blantyre District recognized and personally endorsed the evidence of **U**ndetectable = **U**ntransmittable, they were concerned about how this message might be received by male clients. The result of not communicating the additional benefit of non-infectiousness revealed that PLHIV remained largely uninformed of the advantages of an undetectable VL. These advantages include: not transmitting the virus to others, being able to beget HIV-negative children, being less exposed to stigma and potentially being more at ease in their sexual relationships. Being made aware of these benefits may also improve treatment adherence [20]. The extent to which recent endeavours to train health staff and to vernacularize the U = U message have changed this situation and helped stakeholders to convey the message of non-infectiousness to clients and communities will have to be explored in future studies. Our findings here serve as a reference point. Acknowledging the efforts that Malawi has already made in the field of HIV and in integrating care, we recommend to ensure that health care providers and implementers are fully informed about the interpersonal benefits of a suppressed VL and equipped to pass on this knowledge. While pointing out that additional preventative measures may be necessary to avert possible STIs or pregnancies, we also recommend the consideration of more frequent VL and STI monitoring. This would enable clients to have current information on their VL and STI status so that those with undetectable VLs know that there is no risk of transmitting HIV to a partner, and that any STIs they may have are treated.
